# Analysis of the effectiveness and safety of Jiedu Xiezhuo Yishen Tang in the treatment of gouty arthritis: An observational study

**DOI:** 10.1097/MD.0000000000038582

**Published:** 2024-07-05

**Authors:** Ling He, Yan Peng, Mingqiao Ding

**Affiliations:** a Rehabilitation Department, Wuhan Fifth Hospital, Wuhan, Hubei, China.

**Keywords:** benzbromarone, blood uric acid, gouty arthritis, integrated traditional Chinese and Western medicine, Jiedu Xiezhuo Yishen Tang

## Abstract

This study is for exploring the effectiveness and security of Jiedu Xiezhuo Yishen Tang in the treatment of gouty arthritis. This retrospective study collected 100 patients with gouty arthritis between February 2022 and February 2023. According to the different treatment methods, the data of patients were divided into control group and experimental group. The control group received routine treatment with benzbromarone, while the experimental group received additional treatment with Xuedu Xiezhuo Yishen Tang on the basis of the control group. The evaluation indicators for the effectiveness of treatment include serum levels of 8-hydroxydeoxyguanosine, 3-NT, interleukin-6, interleukin-10, interleukin-1β, tumor necrosis factor-α, C-reactive protein, erythrocyte sedimentation rate, urea nitrogen, creatinine, evaluation of knee joint function and pain level, traditional Chinese medicine syndrome score, and safety evaluation. After treatment, the overall treatment effect of the experimental group reached 98%, while the control group was 78%. After treatment, the differences in various indicators possessed statistical significance (SS) (*P* < .05). In the Lysholm score, the improvement in the experimental group was markedly more excellent than the control group, and the difference possessed SS (*P* < .05). In the NRS score, the experimental group’s NRS score decreased from 8.39 to 1.08 before and after treatment, while the control group only decreased to 3.61. In addition, both groups of patients showed significant improvement in the joint score in the Traditional Chinese medicine syndrome sub-items. The experimental group was able to effectively improve symptoms such as joint pain, joint redness and swelling, joint fever, and limited joint mobility. After treatment, the incidence of adverse reactions in the experimental group was only 8%, while the incidence of adverse reactions in the control group was 24%. After statistical analysis of the incidence of adverse reactions during treatment among the participants, it was found that the difference possessed SS (*P* < .001). The combination treatment of Jiedu Xiezhuo Yishen Tang and benbromarone can effectively improve oxidative stress response and significantly reduce blood uric acid levels. Meanwhile, this combination therapy can effectively inhibit inflammatory reactions, significantly alleviate knee joint pain, and promote the recovery of knee joint function. This treatment regimen has lower toxic side effects and higher safety.

## 1. Introduction

Gouty arthritis is a common inflammatory disease of the joints, with joint inflammation and pain caused by abnormal uric acid metabolism.^[1]^ Long-term hyperuricemia can lead to the deposition of urate salts in joints and soft tissues, causing inflammation, and pain. The treatment of gouty arthritis usually involves prevention and adjuvant treatment by adjusting diet, limiting alcohol consumption, and avoiding fatigue.^[1,2]^ Commonly used uric acid lowering drugs such as benzbromarone and colchicine, although have significant therapeutic effects, have certain toxic side effects. Among them, colchicine can effectively inhibit the aggregation of white blood cells in the site of joint inflammation, weakening the phagocytic effect of white blood cells on uric acid. This can alleviate the inflammatory response caused by local white blood cell damage in the joints. However, high-dose use of colchicine may also cause shock, with main symptoms including hematuria, oliguria or anuria, impaired consciousness or convulsions.^[3]^ Benzbromarone is an effective therapeutic drug for gouty arthritis, characterized by strong uric acid removal, inhibition of uric acid production, and reduction of blood uric acid concentration. This drug effectively alleviates joint inflammation and pain by inhibiting the generation of uric acid and promoting its excretion. However, excessive use of benzbromarone may lead to uric acid like kidney stones, and in severe cases, renal dysfunction or even uremia may occur.^[4]^ In contrast, traditional Chinese medicine (TCM) has rich experience in treating gouty arthritis, and its safety and efficacy have been widely recognized. TCM holds that the pathogenesis of gouty arthritis is mainly caused by factors like damp heat, toxic turbidity, and blood stasis. Therefore, the main direction of TCM treatment is for clearing heat and detoxify, promote dampness and turbidity, and nourish the kidneys and blood. Jiedu Xiezhuo Yishen Tang is a TCM formula, which mainly consists of Chinese herbs such as Poria cocos, Lonicera japonica vine, talcum, and Coix seed. It is believed to have anti-inflammatory, antioxidant, and uric acid metabolism regulating effects. The study aims to explore the effectiveness and safety of Jiedu Xiezhuo Yishen Tang combined with benzbromarone under the gouty arthritis’ treatment. This study evaluated the Jiedu Xiezhuo Yishen Tang combined with benzbromarone under the gouty arthritis’ treatment through a controlled experiment. This is to provide a safer and more effective treatment for gouty arthritis, while also providing a more scientific and reliable basis for TCM treatment of gouty arthritis.

## 2. Research materials and methods

### 2.1. General information

The retrospective study was approved by the Ethics Committee of Wuhan Fifth Hospital. This study collected 100 patients with gouty arthritis from between February 2022 and February 2023. The statistical data of these patients were divided into control group and experimental group according to different treatment methods. with 50 in the experimental group (EG) and 50 in the control group (CG). When selecting research subjects, corresponding inclusion and exclusion criteria were set in the study. The specific inclusion criteria are as follows: age between 18 and 80 years old; diagnosed medically as a patient with gouty arthritis; the patient and their close relatives were informed about the experimental content and signed an informed consent form; the knee joint (KJ) presents with redness, swelling, tenderness, and fever, accompanied by clinical symptoms like general weakness; approved by the hospital ethics committee. The exclusion criteria are as follows: secondary gouty patients caused by renal failure, tumor chemotherapy or radiotherapy, medication, etc; joint injuries caused by diseases such as rheumatoid arthritis, osteoarthritis, or traumatic arthritis; patients with severe cardiovascular, cerebrovascular, liver, kidney, and hematopoietic system diseases; allergy to drugs used in the research institute; pregnant or lactating women; patients with mental disorders and senile dementia; have taken hormone drugs within the past week; patients who do not cooperate with treatment, undergo blood tests, are lost to follow-up, or have incomplete clinical data. The general information of 100 patients with gouty arthritis is showcased in Table 1. Among them, the statistically significant difference (SD) did not exist in the basic information comparison of patients (*P* > .05), and it was comparable.

**Table 1:** General data of 100 patients with gouty arthritis.

Group	Example number	Number of male cases	Number of female cases	Age (yr)	Disease course (yr)
Control group	50	29	21	60.46 ± 7.34	19.73 ± 4.31
Experimental group	50	30	20	60.37 ± 8.64	18.38 ± 4.13
*t*(χ^2^)	0.536			0.439	1.204
*P* value	.495			.634	.246

### 2.2. Treatment method

Before starting treatment, both groups of patients received a low purine diet and drank plenty of water to ensure a daily urine output of 2000 to 3000 milliliters. The CG patients were treated with benzbromarone, manufactured by Shanghai Xinyi Pharmaceutical Co., Ltd., with a dosage of 50 milligrams per day, once a day, taken after meals, for a continuous treatment of 5 weeks. On the basis of the CG, patients in the EG were treated with Jiedu Xiezhuo Yishen Tang. Among them, the formula of Jiedu Xiezhuo Yishen Tang includes 10 g of raw earth, 15 g of Bixie, 30 g of Huangfuzi, 30 g of Chuan Niu Xi, 25 g of Weilingxian, 20 g of Duhuo, 10 g of raw rhubarb, 15 g of Cassia seed, 20 g of Coix seed, 30 g of Yimu grass, 10 g of peach kernel, 20 g of white peony, 15 g of Huangbai, 15 g of Cangzhu, 10 g of Xuanshen, and 10 g of safflower. The patient needs to decoct this decoction daily, take it orally twice, and continue treatment for 5 weeks. During the treatment period, researchers collected specimens from the patient’s fasting venous blood and separated the bleeding serum by centrifugation, which was then stored at −80 °C for future research.

### 2.3. Observation index

During the treatment process, researchers will closely monitor the reactions of both groups of patients and record any adverse reactions (ARs) such as upper abdominal pain, nausea, and vomiting, or elevated transaminases. To evaluate the safety of treatment plans, the study will calculate the incidence of ARs. The calculation method is to divide the number of ARs by the total cases, and then multiply by 100%. The higher the value, the lower the safety of the treatment plan; after starting and ending treatment, researchers extracted blood from the veins of 2 groups of patients and collected urine samples to calculate the overall clinical outcomes of the 2 groups of gouty arthritis patients; to evaluate the oxidative stress status in serum, researchers will measure the levels of 8-hydroxydeoxyguanosine (8-OHdG) and 3-nitrotyrosine (3-NT) in serum using enzyme-linked immunosorbent assay (ELISA); the study aims to determine the concentration of serum uric acid (BUA) using a fully automated biochemical analyzer and uricase assay; for inflammatory factors (IFs) in serum, such as interleukin-6 (IL-6), interleukin-10 (IL-10), interleukin-1β (IL-1β), and tumor necrosis factor-α (TNF-α), researchers quantitatively measured them using ELISA; it evaluates the patient’s levels of C-reactive protein, erythrocyte sedimentation rate (ESR), urea nitrogen, and creatinine by testing blood and urine samples after treatment; the function of the KJ is evaluated using the Lysholm system, which has a maximum score of 100. The higher the score, the more excellent the KJ function; the degree of KJ pain will be evaluated using the Pain Rating Scale (NRS), ranging from 0 to 10 points, with higher scores demonstrating more severe pain; it tests the TCM syndrome scores of patients before and after treatment, including joint redness and swelling, joint pain, joint fever, limited joint movement, thirst, and fever; after the treatment, the researchers evaluated the safety of both groups of patients. During the evaluation process, the main reference was made to the 2022 Guidelines for Clinical Research of New Chinese Medicines (Trial), and the patient’s digestive disorders and abdominal pain before and after treatment were observed. In addition, other possible ARs were observed, such as gastrointestinal discomfort, bloating, vomiting, diarrhea, bloody and black stools, as well as safety indicators such as liver and kidney function.

### 2.4. Statistical analysis

This study analyzed the test data in SPSS 20.0 software. For quantitative data, the study used mean ± standard deviation $\bar{x}\pm s$ to represent it, and to compare these data, a *t* test was used. For counting data, the study mainly used χ^2^-test. All percentages are expressed in (%). The comparison of 2 sets of level data is conducted using rank sum test. At the same time, the study sets *P* < .05 a*s* the threshold for statistically SDs.

## 3. Results

### 3.1. Clinical efficacy comparison

Table 2 shows the total effective rate (ER) of clinical treatment in 100 patients with gouty arthritis. According to Table 2, the total ER of the CG after corresponding treatment is 39 cases, accounting for 78% of the total. The total ER of the EG was as high as 98%, with only 1 patient being ineffective. After rank sum test, *Z* = 1.634, *P* = .105, and the difference did not possess statistical significance (SS).

**Table 2:** Clinical treatment total ER of 100 patients with gouty arthritis in the EG.

Group	Number of cases		Proportion	
	CG	EG	CG	EG
Invalid	11	1	22%	2%
Effective	8	7	16%	14%
Significant effect	12	12	24%	24%
Heal	19	30	38%	60%
Total effective rate	39	49	78%	98%

### 3.2. Comparison of serum oxidative stress indicators and serum uric acid levels

**Figure 1.**
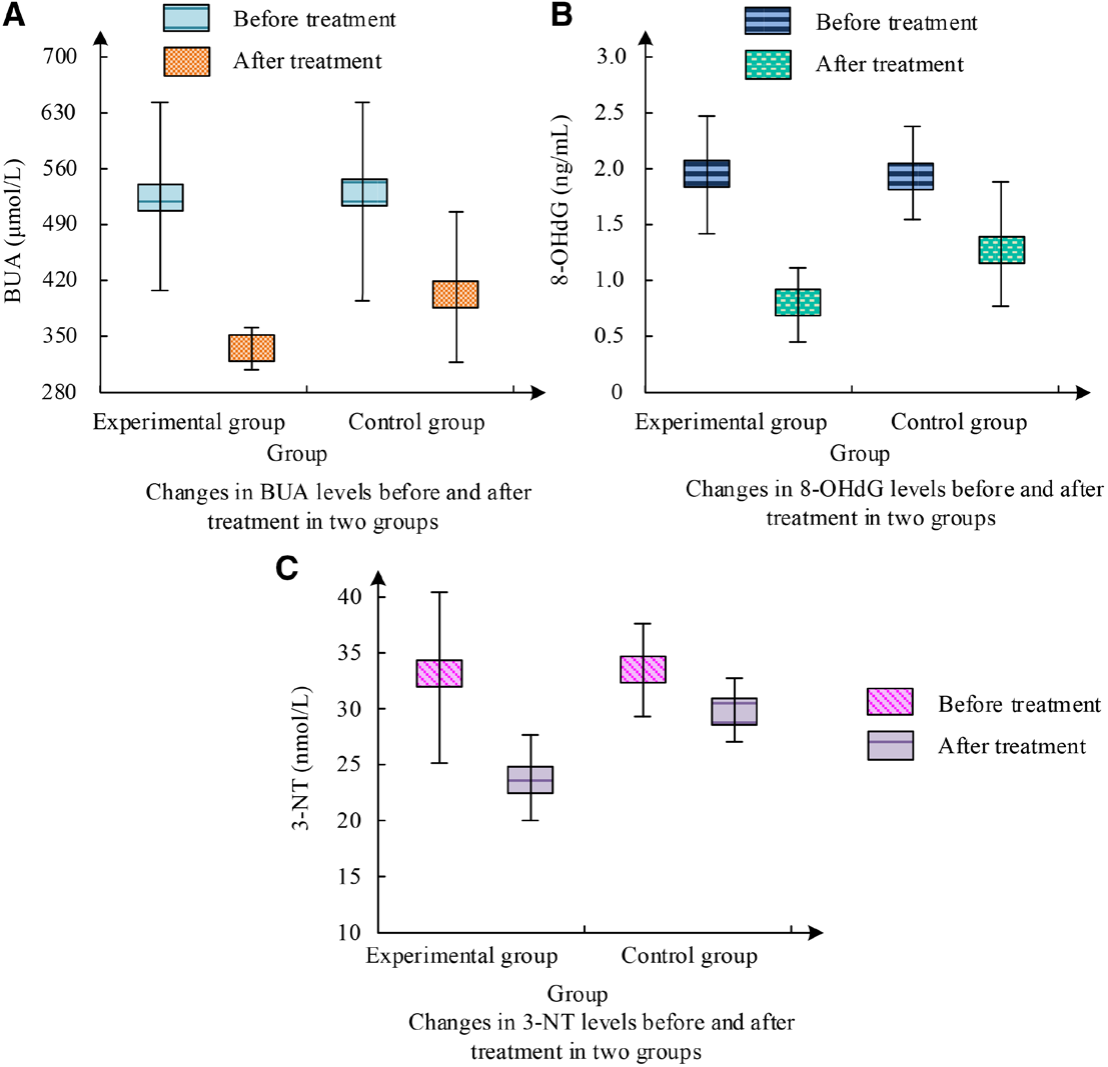
BUA, 8-OHdG, 3-NT levels before and after treatment.

Figure 1 shows the comparison results of BUA, 8-OHdG, and 3-NT levels between 2 groups of patients before and after treatment. As shown in Figure 1A, the BUA level in the EG decreased from 524.03 μmol/L before treatment to 320.58 μmol/L, with a significant decrease compared to the CG. According to Figure 1B, the 8-OHdG level in the EG decreased from 1.93 to 0.77 ng/mL, while the CG had a high 8-OHdG level of 1.23 ng/mL after treatment. As shown in Figure 1C, the 3-NT level in the EG decreased from 33.56 to 23.68 nmol/L, which was significantly better than the CG. Compared to the CG, the treatment effect of the EG is more significant.

The *t* values and *P* values of various indicators before and after treatment for 2 groups of patients are shown in Table 3. According to Table 3, before treatment, no SDs were found in the serum levels of 8-OHdG, 3-NT, and BUA among the participants (*P* > .05). After treatment, these indicators decreased in both groups of patients. The decrease in the EG was more significant compared to the CG, with a *P* value < .05, which possesses SS.

**Table 3:** The *t* values and *P* values of BUA, 8-OHdG, 3-NT before and after treatment.

Index	BUA (μmol/L)		8-OHdG (ng/mL)		3-NT (nmol/L)	
	Before treatment	After treatment	Before treatment	After treatment	Before treatment	After treatment
*t* value	0.136	3.723	0.048	4.692	0.048	2.624
*P* value	.812	<.001	.934	<.001	.916	.005

### 3.3. Comparison of serum IF results

**Figure 2.**
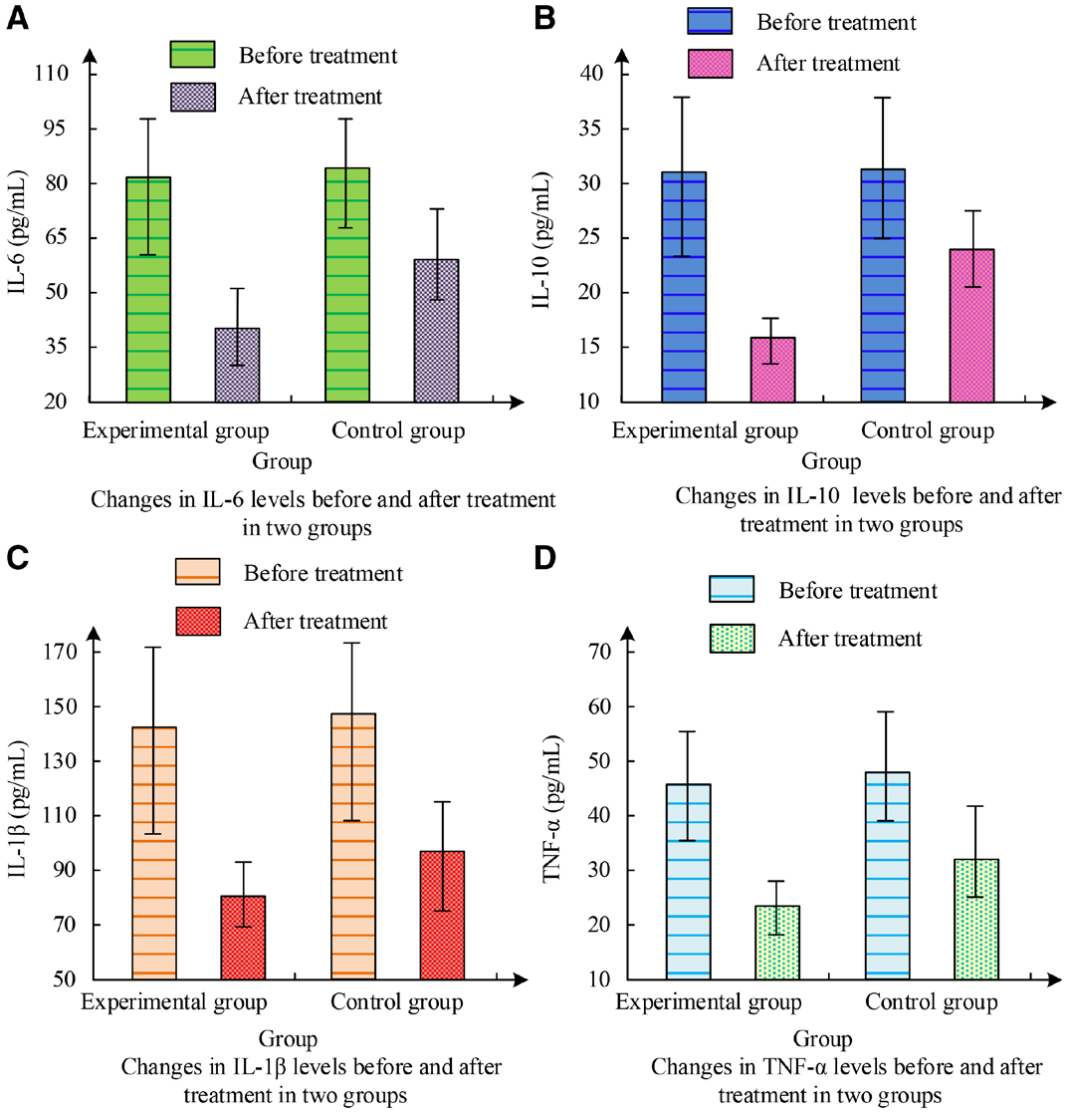
Serum levels of IL-6, IL-10, IL-1β, and TNF-α in both groups.

Figure 2 shows the changes in these serum IFs before and after treatment. Figure 2A shows that the average value of IL-6 in the EG before treatment was 82.01 pg/mL, which decreased to 41.25 pg/mL after treatment. The average value of IL-6 in the CG after treatment was as high as 59.67 pg/mL. According to Figure 2B, the average value of IL-10 in the EG before treatment was 31.14 pg/mL, which decreased to 16.92 pg/mL after treatment, significantly better than the CG. Figure 2C shows that the average IL-1β levels of the 2 groups before treatment were around 144 pg/mL. After treatment, the EG decreased to 79.14 pg/mL, while the CG reached as high as 97.58 pg/mL. As shown in Figure 2D, the EG showed a significant decrease in TNF-α levels after treatment compared to the CG. The EG has a better therapeutic effect on gouty arthritis.

The *t* values and *P* values of various indicators before and after treatment for the 2 groups of patients are shown in Table 4. Table 4 shows that before treatment, the SD did not exist in these serum IFs among the participants, and no SD was found in statistical comparison (*P* > .05). After corresponding treatment, these serum IFs showed a downward trend, and the decrease in the EG was more significant compared to the CG, with a statistical difference (*P* < .05).

**Table 4:** The t-values and *P*-values of IL-6, IL-10, IL-1β, and TNF-α before and after treatment.

Index	IL-6 (pg/mL)		IL-10 (pg/mL)		IL-1β (pg/mL)		TNF-α (pg/mL)	
	Before treatment	After treatment	Before treatment	After treatment	Before treatment	After treatment	Before treatment	After treatment
*t* value	0.045	4.358	0.139	5.035	0.072	2.674	0.087	5.068
*P* value	.924	<.001	.943	<.001	.993	.011	.910	<.001

### 3.4. Comparison of C-reactive protein, ESR, urea nitrogen, and creatinine indicators

Table 5 shows the levels of C-reactive protein, ESR, and creatinine in patients after treatment. Table 5 shows that after treatment, the C-reactive protein level in the EG decreased to 28.61 μmol/L, while the C-reactive protein level in the CG only decreased to 43.41 μmol/L. Meanwhile, the ESR level in the EG decreased to 61.48 mg/L after treatment, while in the CG it reached as high as 61.48 mg/L. In addition, the urea nitrogen level in the EG decreased to 6.43 mm/h after treatment, significantly lower than that in the CG. Finally, the creatinine level in the EG decreased to 95.48 μmol/L, while in the CG it reached as high as 123.84 μmol/L. At the same time, this indicates that after treatment, the decline in various indicators of the EG was better than that of the CG. The difference in changes among the participants possesses SS (*P* < .05).

**Table 5:** The *t* values and *P* values of C-reactive protein, blood sedimentation, urea nitrogen, and creatinine index after treatment ($\bar{x}\pm s$).

Index	C-reactive protein (μmol/L)	Blood sedimentation (mg/L)	Urea nitrogen (mm/h)	Creatinine index (μmol/L)
CG	43.41 ± 23.18	61.48 ± 17.05	11.54 ± 1.38	123.84 ± 14.09
EG	28.61 ± 11.68	46.11 ± 12.08	6.43 ± 1.25	95.48 ± 13.67
*t* value	2.619	3.576	10.336	7.037
*P* value	.007	.001	.000	.000

### 3.5. Comparison of KJ function and pain severity scores

**Figure 3.**
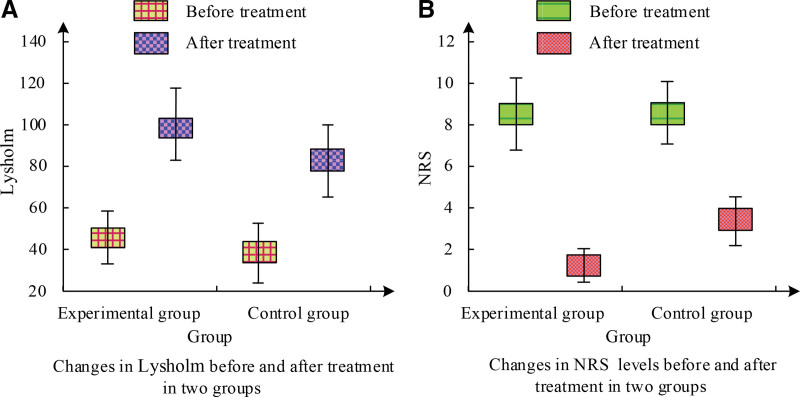
Results of the Lysholm and NRS scores before and after treatment.

Figure 3 shows the Lysholm and NRS scores before and after treatment. Figure 3A shows that the Lysholm score of the EG increased from 44.38 to 99.27 before and after treatment, which markedly exceeds the score of the CG. As shown in Figure 3B, the NRS score of the EG decreased from 8.39 to 1.08 before and after treatment, while the CG only decreased to 3.61. The EG showed better recovery of KJ function after treatment, and at the same time, their pain level was lower.

The *t* values and *P* values of Lysholm and NRS scores before and after treatment for 2 groups of patients are shown in Table 6. Table 6 shows that before treatment, there was no SD in Lysholm and NRS scores among the participants (*P* > .05). After corresponding treatment, there was a statistically SD (*P* < .05) between the 2.

**Table 6:** The *t* values and *P* values of Lysholm and NRS before and after treatment.

Index	Lysholm		NRS	
	Before treatment	After treatment	Before treatment	After treatment
*t* value	0.036	2.139	0.384	10.674
*P* value	.988	.0033	.709	<.001

### 3.6. TCM syndrome points

**Figure 4.**
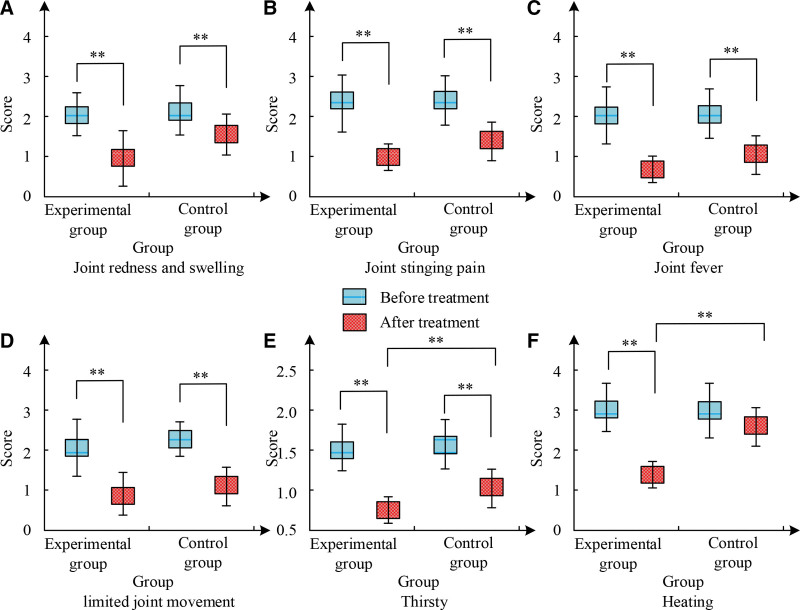
The TCM syndrome score results before and after treatment. TCM = Traditional Chinese medicine.

Figure 4 shows the TCM syndrome integration results of patients before and after treatment. After treatment, the significant improvement existed in the joint integral of the TCM syndrome sub items in both groups of patients. There was a SD in various indicators between the EG and the CG before and after treatment (*P* < .01). Relative to the CG, the EG after treatment showed significant improvement in symptoms such as fever and thirst, with a statistically SD (*P* < .01). Both treatment plans were effective in improving symptoms such as joint pain, joint redness and swelling, joint fever, and limited joint mobility, but the treatment effect in the EG was more significant. This indicates that the treatment methods in the EG are more practical and applicable.

### 3.7. Security comparison

The safety comparison results among the participants before and after treatment are shown in Table 7. According to Table 7, after treatment, only 4 patients in the EG experienced diarrhea, which was improved by adjusting the dosage of TCM. In the CG, 8 patients experienced diarrhea, 2 experienced vomiting and flushing, 1 experienced abdominal distension and pain, and 1 experienced liver and kidney dysfunction. The incidence of ARs in the EG was only 8%, while the incidence of ARs in the CG was 24%. The incidence of ARs during treatment was tested for the participants, and the outcomes showcased *P* < .05, which possesses SS. This indicates that the toxic side effects of the EG are lower than those of the CG.

**Table 7:** Comparison of safety before and after treatment.

Group	Experimental group	Control group
Total number of cases	50	50
Diarrhoea	4	8
Vomiting, plain water	0	2
Abdominal swelling pain	0	1
Abnormal liver and kidney function	0	1
Total number of cases	4	12
Total incidence	8%	24%

## 4. Discussion

Gouty arthritis is a chronic metabolic disease from disturbances in uric acid metabolism. Uric acid is a purine metabolite. Under normal circumstances, uric acid in the human body is excreted through the kidneys and maintained within a certain concentration range. However, when the production rate of uric acid is too fast or the excretion rate is too slow, uric acid will accumulate in the body, forming urate salt crystals, which can result in the occurrence of gouty arthritis. The pathogenesis of gouty arthritis is mainly related to the inflammatory response caused by uric acid crystals.^[5]^ When urate deposits and crystallizes in the joints, it activates inflammatory cells, releases pro-inflammatory mediators, and causes an inflammatory response in the surrounding tissues of the joints. These inflammatory reactions manifest as joint redness, swelling, heat, and pain, and in severe cases can lead to limited joint function. The incidence factors of gouty arthritis are diverse, including genetic factors, lifestyle, and dietary habits. Genetic factors are one of the important influencing factors of gout. If there is a family history of gout, the individual’s risk of developing the disease will increase.^[6]^ In addition, dietary habits are also an important factor in the onset of gout. Consuming high purine foods such as animal organs, seafood, and red meat can increase the production and accumulation of uric acid, thereby increasing the risk of illness. In terms of lifestyle, lack of exercise, obesity, excessive alcohol consumption, etc. are also closely related to the occurrence of gout. The clinical manifestations of gouty arthritis mainly include acute attacks and chronic course.^[7]^ During acute attacks, patients often experience sudden symptoms of joint redness, swelling, heat, and pain, which are severe and often occur in the large toe joint of the foot. During the chronic course of the disease, the patient’s joint inflammation relapses repeatedly, and the interval between episodes gradually shortens, leading to limited joint function.^[8]^ In TCM, the treatment of gouty arthritis follows the principles of promoting blood circulation and unblocking collaterals, dispelling wind and dampness, removing turbidity and stasis, and clearing heat and relieving pain.^[9–12]^ Based on this concept, research is being conducted on the addition of the formula of Du Xie Zhuo Yi Shen Tang in addition to taking Western medicine. In this formula, Duhuo and Cangzhu have the effects of warming meridians, dispersing cold, removing dampness, and relieving pain. Weiling Xian can dispel wind, remove dampness, unblock meridians, and relieve pain. Cassia seed has beneficial renal effects. Rhubarb and Fructus Rehmanniae can clear heat, detoxify, dispel wind and dampness. Coix seed can achieve invigorating the spleen and dispelling dampness, relaxing tendons and removing dampness, clearing heat and expelling pus. White peony can nourish blood, soften the liver, and relieve pain. Huangbai can achieve clearing heat, drying dampness, purging fire, and detoxifying. Chuan Niu Xi can promote blood circulation and meridian circulation, dispel wind and remove dampness. Bi Xie can achieve dispelling wind and removing dampness and turbidity. Xuanshen and Shengdi can achieve clearing heat and cooling blood. Peach kernels, safflower, and motherwort can promote blood circulation and remove blood stasis. The combined use of these drugs can achieve clearing heat and detoxifying, promoting blood circulation and removing stasis, clearing heat and dispelling dampness.^[13–15]^

This study indicates that the combination of benzbromarone and Jiedu Xiezhuo Yishen Tang has a better therapeutic effect relative to single Western medicine treatment. Among them, in the comparative experiment of the total ER of clinical treatment, the CG had a total ER of 39 cases after corresponding treatment, accounting for 78% of the total. The total ER of the EG was as high as 98%, with only 1 patient being ineffective. This is similar to the research results of Xie et al. Meanwhile, the BUA level in the EG decreased from 524.03 μmol/L before treatment to 320.58 μmol/L, the 8-OHdG level decreased from 1.93 to 0.77 ng/mL, and the 3-NT level decreased from 33.56 to 23.68 nmol/L. The changes in these indicators were significantly better than those in the CG. In the study by Huang et al^[16]^, it was also found that the combination of TCM decoction treatment had a more significant effect in the group. In the serum IF experiment, the average value of IL-6 in the EG before treatment was 82.01 pg/mL, which was reduced to 41.25 pg/mL after treatment. IL-10 was 31.14 pg/mL before treatment and decreased to 16.92 pg/mL after treatment, markedly more excellent than the CG. IL-1β was around 144 pg/mL before treatment, but after treatment, it decreased to 79.14 pg/mL in the EG and 97.58 pg/mL in the CG. The EG showed a significant decrease in TNF-α levels after treatment compared to the CG. Guo et al^[17]^ have also obtained similar results. After treatment, the C-reactive protein level in the EG decreased to 28.61 μmol/L, ESR decreased to 61.48 mg/L, urea nitrogen level decreased to 6.43 mm/h, and creatinine level decreased to 95.48 μmol/L. Ren et al^[18]^ found that combining TCM treatment can significantly reduce these indicators. In the KJ function and pain score experiment, the Lysholm score of the EG increased from 44.38 to 99.27 after treatment, markedly exceeding the score of the CG. In addition, the NRS score of the EG decreased from 8.39 to 1.08 before and after treatment, while the CG only decreased to 3.61. These outcomes showcase that the EG possessed more excellent recovery of KJ function after treatment, and their pain level was also lower. The study by Zhou et al^[19]^ shows that traditional Chinese and Western medical methods have a better promoting effect on the recovery of KJ function. In the evaluation of TCM syndrome integration, both groups of patients showed significant improvement in joint single item integration. There was a SD in various indicators before and after treatment between the EG and the CG (*P* < .01). Compared with the CG, the EG after treatment showed significant improvement in symptoms such as fever and thirst, with a statistically SD (*P* < .01). Both treatment plans were effective in improving symptoms such as joint pain, joint redness and swelling, joint fever, and limited joint mobility, but the treatment effect in the EG was more significant. This is similar to the research results of Li et al.^[20]^ In the safety comparison experiment, after treatment, only 4 patients in the EG experienced diarrhea, while 8 patients in the CG experienced diarrhea. Some patients also experienced ARs such as vomiting, flushing, abdominal pain, and abnormal liver and kidney function. The incidence of ARs in the EG was only 8%, while the incidence of ARs in the CG was 24%. The toxicity and side effects of the EG were lower than those of the CG. The results of researchers such as Bibi et al^[21]^ show that the toxic side effects of integrated traditional Chinese and Western medicine treatment are below single Western medicine treatment. This study still has limitations: the sample size of this study is small; the follow-up time of patients is small, and the follow-up time should be increased; as a retrospective study, confounding bias and selection bias still exist in the selection of patient data. This is a retrospective study, but it does not affect the accuracy of the study. In the future, we plan to conduct a multicenter RCT study with extended follow-up to observe the long-term efficacy of Jiedu Xiezhuo Yishen Tang in patients.

In summary, the combination therapy can effectively treat patients with gouty arthritis. Among various treatment methods, the combination of Jiedu Xiezhuo Yishen Tang and benbromarone has significant advantages. Jiedu Xiezhuo Yishen Tang is a treasure of TCM in China, which can achieve clearing heat and detoxifying, promoting diuresis and turbidity, and nourishing the kidneys and liver. Modern pharmacological research confirmed that Jiedu Xiezhuo Yishen Tang can decrease blood uric acid levels, strengthen kidney function, and have anti-inflammatory and analgesic effects. As a new type of nonsteroidal anti-inflammatory drug, benzbromarone had good anti-inflammatory, analgesic, and antipyretic effects. However, while receiving the treatment of Jiedu Xiezhuo Yishen Tang combined with benzbromarone, patients also needed to pay attention to dietary regulation, maintain good sleep habits, and strengthen exercise. This study did not consider the external conditions of patients, and further optimization can be carried out.

## Author contributions

**Conceptualization:** Ling He, Yan Peng.

**Data curation:** Ling He, Yan Peng, Mingqiao Ding.

**Formal analysis:** Ling He, Yan Peng, Mingqiao Ding.

**Investigation:** Ling He, Mingqiao Ding.

**Methodology:** Ling He, Yan Peng, Mingqiao Ding.

**Supervision:** Ling He, Yan Peng.

**Writing – original draft:** Ling He, Yan Peng.

**Writing – review & editing:** Ling He, Yan Peng, Mingqiao Ding.

**Funding acquisition:** Yan Peng.

**Validation:** Yan Peng, Mingqiao Ding.
